# Association of *IL1A *and *IL1B *loci with primary open angle glaucoma

**DOI:** 10.1186/1471-2350-11-99

**Published:** 2010-06-19

**Authors:** Suddhasil Mookherjee, Deblina Banerjee, Subhadip Chakraborty, Antara Banerjee, Indranil Mukhopadhyay, Abhijit Sen, Kunal Ray

**Affiliations:** 1 Molecular & Human Genetics Division, Indian Institute of Chemical Biology, Council of Scientific & Industrial Research, Kolkata, India; 2 Human Genetics Unit, Indian Statistical Institute, Kolkata, India; 3 Dristi Pradip, Jodhpur Park, Kolkata, India

## Abstract

**Background:**

Recent studies suggest that glaucoma is a neurodegenerative disease in which secondary degenerative losses occur after primary insult by raised Intraocular pressure (IOP) or by other associated factors. It has been reported that polymorphisms in the *IL1A *and *IL1B *genes are associated with Primary Open Angle Glaucoma (POAG). The purpose of our study was to investigate the role of these polymorphisms in eastern Indian POAG patients.

**Methods:**

The study involved 315 unrelated POAG patients, consisting of 116 High Tension Glaucoma (HTG) patients with intra ocular pressure (IOP) > 21 mmHg and 199 non-HTG patients (presenting IOP < 20 mmHg), and 301 healthy controls from eastern India. Genotypes were determined by polymerase chain reaction and restriction digestion for three single nucleotide polymorphisms (SNPs): *IL1A *(-889C/T; rs1800587), *IL1B *(-511C/T; rs16944) and *IL1B *(3953C/T; rs1143634). Haplotype frequency was determined by Haploview 4.1 software. The association of individual SNPs and major haplotypes was evaluated using chi-square statistics. The p-value was corrected for multiple tests by Bonferroni method.

**Results:**

No significant difference was observed in the allele and genotype frequencies for *IL1A *and *IL1B *SNPs between total pool of POAG patients and controls. However, on segregating the patient pool to HTG and non-HTG groups, weak association was observed for *IL1A *polymorphism (-889C/T) where -889C allele was found to portray risk (OR = 1.380; 95% CI = 1.041-1.830; p = 0.025) for non-HTG patients. Similarly, 3953T allele of *IL1B *polymorphism (+3953C/T) was observed to confer risk to HTG group (OR = 1.561; 95% CI = 1.022-2.385; p = 0.039). On haplotype analysis it was observed that TTC was significantly underrepresented in non-HTG patients (OR = 0.538; 95% CI = 0.356- 0.815; p = 0.003) while TCT haplotype was overrepresented in HTG patients (OR = 1.784; 95% CI = 1.084- 2.937; p = 0.022) compared to control pool. However, after correction for multiple tests by Bonferroni method, an association of only TTC haplotype with non-HTG cases sustained (p_corrected _= 0.015) and expected to confer protection.

**Conclusion:**

The study suggests that the genomic region containing the *IL1 *gene cluster influences the POAG pathogenesis mostly in non-HTG patients in eastern India. A similar study in additional and larger cohorts of patients in other population groups is necessary to further substantiate the observation.

## Background

Glaucoma is a heterogeneous group of optic neuropathy, characterized by typical visual field loss, often associated with elevated intra ocular pressure. It affects over 60 million people worldwide and is the second largest cause of blindness after cataract [1]. Among different subtypes of glaucoma, Primary Open Angle Glaucoma (POAG) is the most frequently occurring subtype. Till date 25 loci have been implicated in the pathogenesis of POAG with three underlying genes, i.e. *Myocilin *[2], *Optineurin *[3] and *WDR36 *[4]. Recent studies suggest that POAG is caused mainly by genetic predisposition and interaction with other risk factors. It is estimated that 72% of all POAG cases represent the inherited and familial form of the disease that does not show a clear pattern of Mendelian inheritance [5].

POAG results from progressive excavation of the optic disc with corresponding loss of vision by raised Intraocular pressure (IOP) or by other associated factors. However, a substantial number of POAG patients have normal IOP - a condition referred to as normal tension glaucoma (NTG). The main aqueous outflow pathway, controlling IOP, consists of series of endothelial cell-lined channels in the angle of the anterior chamber such as the trabecular meshwork (TM), Schlemm's canal, the collector channels and the episcleral venous system. Accumulation of extraneous materials or cells within the TM, alterations in the cell structure, accelerated TM cell death and collapse of trabecular beams as a result of oxidative stress, vascular dysregulation and aging causes reduced outflow in open angle glaucoma. The cells surviving the sublethal injury tend to mount a protective response involving the expression of new genes [6].

It has been suggested that, in addition to other contributing factors, immune system in the body may have a role in neuro-degeneration in glaucoma [7]. For example, antibody levels against some of the self proteins [8,9] e.g. anti-NSE antibody [10], antibodies to heat shock proteins [11] and beta 2 glycoproteins [12] have been reported to be significantly higher in glaucoma patients. It has been reported that Interleukin-1 (IL1), an inflammatory cytokine, is involved in ischemic and excitotoxic damage in the retina [6]. Unlike the normal TM cells, glaucomatous cells show endogenous expression of IL1. *In vitro *experiments show that a stress response specific to the aqueous outflow pathway is activated in the TM cells and controlled by an Interleukin-1 (IL1) autocrine feedback loop through transcription factor NF-kappaB [6]. It has also been suggested that the chronic activation of the stress response in the aqueous outflow pathways might initiate and exacerbate high tension glaucoma (HTG) [6].

Shaftel et al have demonstrated that IL1 protein promotes the development of β-amyloid deposits in Alzheimer's patients [13]. Similar β-amyloid plaques are found in the Retinal Ganglion Cells (RGC) of mouse with experimental glaucoma. Drugs which work to prevent this buildup of the β-amyloid protein in Alzheimer brains can be used to treat glaucoma in animal models [14]. Interestingly, studies conducted on Japanese and German population groups suggest a high frequency of POAG in patients with Alzheimer's disease (AD) [15,16]. Both AD and glaucoma show similar neuronal degeneration. Moreover, patients with AD shows high level of axonal degeneration of the optic nerve and retinal cell damage, especially ganglion cells [17,18]. The ε4 allele of *APOE *has been shown to be significantly over expressed in AD and glaucoma patients, though the contribution remains controversial [19,20]. Thus glaucoma may be viewed as a neuro-degenerative disorder similar to that of Alzheimer's with common genetic risk factors, mechanisms and pathways.

Several SNPs in the *IL1 *gene cluster (i.e. *IL1A *-889C/T; *IL1B *-511 C/T & 3953C/T) have been reported to be associated with Alzheimer's disease [21,22]. Recently these SNPs have also been examined for association with POAG through multiple studies in Chinese population [23-27]. Two independent studies by Wang *et al *[25] and Lin *et al *[24] have claimed association of POAG with *IL1A *(-889 C/T) and *IL1B *(3953 C/T) SNPs, respectively. However, a recent study by How *et al *[23] reported a lack of association of these polymorphisms with the disease.

**Figure 1:**
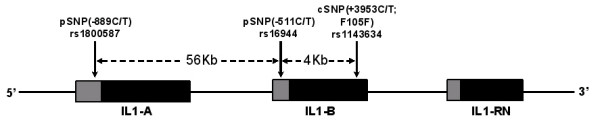
**Location of the SNPs in *IL1 *gene cluster *IL1 alpha*, *IL1 beta*, *IL1 receptor antagonist (RN)***. The promoter regions and coding sequences of the genes are shown in light and dark shaded boxes. The dbSNP refrence SNP ID for each SNP is provided. rs1800587 and rs16944 are located in the promoter (p) region of *IL1A *and *IL1B*, respectively while rs1143634 represents a synonymous SNP (F105F) in the coding (c) sequence of *IL1B *gene. The genomic distances between the SNPs are also shown.

In the context of developing information on the potential involvement of the *IL1 *gene cluster in POAG, we aimed to investigate the role of this genomic region using the well-studied three SNPs (*IL1A *-889C/T, *IL1B *-511C/T, *IL1B *+3953C/T) (Figure 1) in eastern Indian patient cohort.

## Methods

### Selection of study subjects

The patients with POAG and control subjects were recruited for the study from Dristipradip Eye Clinic, Kolkata. Individuals in both the cohorts are inhabitants of Kolkata, West Bengal (eastern India), speak Bengali language, and belong to the Indo-European linguistic group. The patient cohort consisted of 315 POAG patients consisting of 116 patients with presenting IOP > 21 mmHg, considered as high tension glaucoma (HTG) cases and 199 non-HTG patients with presenting IOP < 20 mmHg.

Diagnosis of POAG involved clinical, ocular and systemic examinations. Intraocular pressure (IOP) was measured by Goldmann applanation tonometry (Haag-Streit USA Inc., Mason, OH) followed by pachymetry (Ocuscan A, Alcon, Texas, USA). A Goldman 3-mirror gonioscope (Ocular Instrument, Bellevue, WA) was used to assess the angles of the anterior chamber and the optic disc. The optic disc was also evaluated with a +78D lens. Automated threshold field analysis was done using the Humphrey Field Analyzer II (Carl Zeiss, Dublin, CA). The retinal nerve fiber layer (RNFL) was investigated by Scanning Laser Polarimetry (SLP) with variable corneal compensation technique (GDx-Vcc, Carl Zeiss, Dublin, CA).

An increased intraocular pressure above 21 mmHg, significant cupping of the optic disc (> 0.7) with or without peripapillary changes and the presence of clinically open angle (angle of the anterior chamber) on Gonioscopy raised the suspicion of POAG, which was confirmed by typical reproducible visual field changes, viz. arcuate, Bjerrum, Seidel, paracentral and annular scotoma and nasal steps and Scanning Laser Polarimetry for RNFL analysis (Nerve fibre Indicator > 30). The pre-perimetric cases were identified by RNFL analysis. These patients were categorised as the HTG patients. There was also another group of individuals, the non-HTG patients, who had an IOP less than 20 mmHg on presentation but had cupping of the optic disc, RNFL loss diagnosed by SLP and visual field changes characteristic of POAG. In each case the IOP has been corrected for central corneal thickness (CCT). Thus, the patient pool consisted of 315 adult onset open angle glaucoma cases. The age at diagnosis ranged from 42 to 88 years, with a mean ± standard deviation of 64 ± 10 years. However, individuals with any history of inflammation or ocular trauma (past and present) and ocular hypertension were excluded from this study.

In this study, 301 controls were recruited following the criteria which include: age > 40 years (mean age ± SD, 55.7 ± 10.7 years), without any family history of glaucoma or ocular hypertension, IOP less than 20 mmHg in both eyes in at least last two check ups, CCT greater than 500 μm in both eyes, no visual field defect, normal Scanning Laser Polarimeter parameters (i.e. a good yellowish bow type scan pattern, deviation map within normal limit, a good double hump pattern in conduction map, TSNIT parameters within normal limit, Nerve Fibre Indicator < 30 for both eyes), cup discs were physiological and similar in both eyes, cup to disc ratio < 0.2, no defect in disc rim or margin and no sphincter haemorrhage around the disc. Individuals with high myopia, diabetes and hypertension were excluded from the control group.

### Collection of blood samples and genomic DNA preparation

Eight milliliters of peripheral blood was collected with EDTA from the POAG patients and normal individuals with their written consent. Genomic DNA was prepared from fresh whole blood using the PAX gene blood DNA isolation kit (Qiagen, Hilden, Germany). The DNA was dissolved in TE (10 mM Tris-HCl, 1 mM EDTA, pH 8.0). The study protocol adhered to the tenets of the Declaration of Helsinki and was approved by the Institutional Review Board.

### Genotyping

Genotyping was done by polymerase chain reaction and restriction digestion (PCR-RFLP). All the PCR reaction was carried out in 20 μl reaction volume using 80 ng of total genomic DNA with Ex Prime Taq Premix (GeNet Bio, South Korea) with specific primers for *IL1A *(-889C/T), *IL1B *(-511C/T) and *IL1B *(3953C/T) (Integrated DNA Technologies, Coralville, Iowa, USA) as described previously [25,27]. The PCR conditions were as follows: an initial denaturation at 95°C for 4 mins followed by 30 cycles of 30 secs of denaturation at 95°C, 30 secs of annealing at 58-60°C and 30 secs of extension at 72°C, with a final extension at 72°C for 4 mins. All the PCR products were detected on a 6% polyacrylamide gel with ethidium-bromide staining. The PCR products for both *IL1A *and *IL1B *were subjected to restriction digestion with appropriate enzymes from NEB (New England Biolabs Inc. Beverly, MA) for 3 hrs at optimum temperatures as described previously [25,27]. The digested products were analyzed on a 6% polyacrylamide gel and the alleles were scored as described in earlier studies [25,27].

### Bioinformatics and Statistical analysis

Haplotypes and their frequencies were determined for comparison between patients and controls using Haploview 4.1 software http://www.broad.mit.edu/mpg/haploview[28]. The allele frequencies of the SNP and haplotypes were compared between patients and controls using chi square test. The p-values were corrected for multiple testing by Bonferroni method.

## Results

### Lack of association between *IL1A *and *IL1B *polymorphisms and POAG

On genotyping 315 patients for three selected SNPs, *IL1A *(-889C/T), *IL1B *(-511C/T) and *IL1B *(3953C/T), no significant association was observed with the POAG. However, on further sub-dividing the patients according to their presenting IOP, i.e. IOP > 21 mmHg (HTG) and the IOP < 20 mmHg (non-HTG), marginal associations were observed in non-HTG patient group and HTG patient group with *IL1A *(-889C) allele (OR = 1.380; 95% CI = 1.041- 1.830; p = 0.025) and *IL1B *(+3953T) allele (OR = 1.561; 95% CI = 1.022- 2.385; p = 0.039), respectively. However, the observed association was nullified after Bonferroni correction for multiple tests (Table 1). Therefore, our data did not unequivocally suggest having an association with the three SNPs examined.

**Table 1:** Allele frequencies of SNPs in *IL-1 *gene cluster in POAG patients and controls

SNP (Gene)	Allele	Patient Subgroup	Patient (n)	Control (n)	p-value	Adjusted p-Value*	OR (95% CI)
-889C/T rs1800587 (*IL-1A*)		POAG	0.71 (448)		0.204	-	-
	C	Non-HTG	0.74 (296)	0.68 (408)	0.025	0.075	1.380(1.041-1.830)
		HTG	0.66 (152)		0.534	-	-
	
		POAG	0.29 (182)		0.204	-	-
	T	Non-HTG	0.26 (102)	0.32 (194)	0.025	0.075	0.725(0.547-0.961)
		HTG	0.34 (80)		0.534	-	-

-511C/T rs16944 (*IL-1B*)		POAG	0.44 (276)		0.114	-	-
	C	Non-HTG	0.43 (173)	0.39(237)	0.197	-	-
		HTG	0.44 (103)		0.185	-	-
	
		POAG	0.56 (354)		0.114	-	-
	T	Non-HTG	0.57 (225)	0.61(365)	0.197	-	-
							
		HTG	0.56 (129)		0.185	-	-

+3953C/T rs1143634 (*IL-1B*)		POAG	0.85 (538)		0.102	-	-
	C	Non-HTG	0.87 (345)	0.89 (533)	0.380	-	-
		HTG	0.83 (193)		0.039	0.117	0.641(0.419-0.979)
	
		POAG	0.15 (92)		0.102	-	
	T	Non-HTG	0.13 (53)	0.11(69)	0.380	-	-
							
		HTG	0.17 (39)		0.039	0.117	1.561(1.022-2.385)

* P-values were adjusted by Bonferroni method.

The location of the SNP in the genomic region and corresponding dbSNP reference ID is furnished. None of the 3 SNPs was significantly associated with the entire POAG patient cohort or when it is subdivided to two groups: HTG (IOP > 21 mmHg) and non-HTG (IOP < 20 mmHg). The numbers in parenthesis next to 'Allele frequency' represent numbers of chromosomes in the group. The weak association of *IL1A *(-889C/T) and *IL1B *(+3953C/T) polymorphisms were nullified after Bonferroni adjustments for multiple tests.

### *IL1 *haplotype distribution among patient and control group

**Figure 2:**
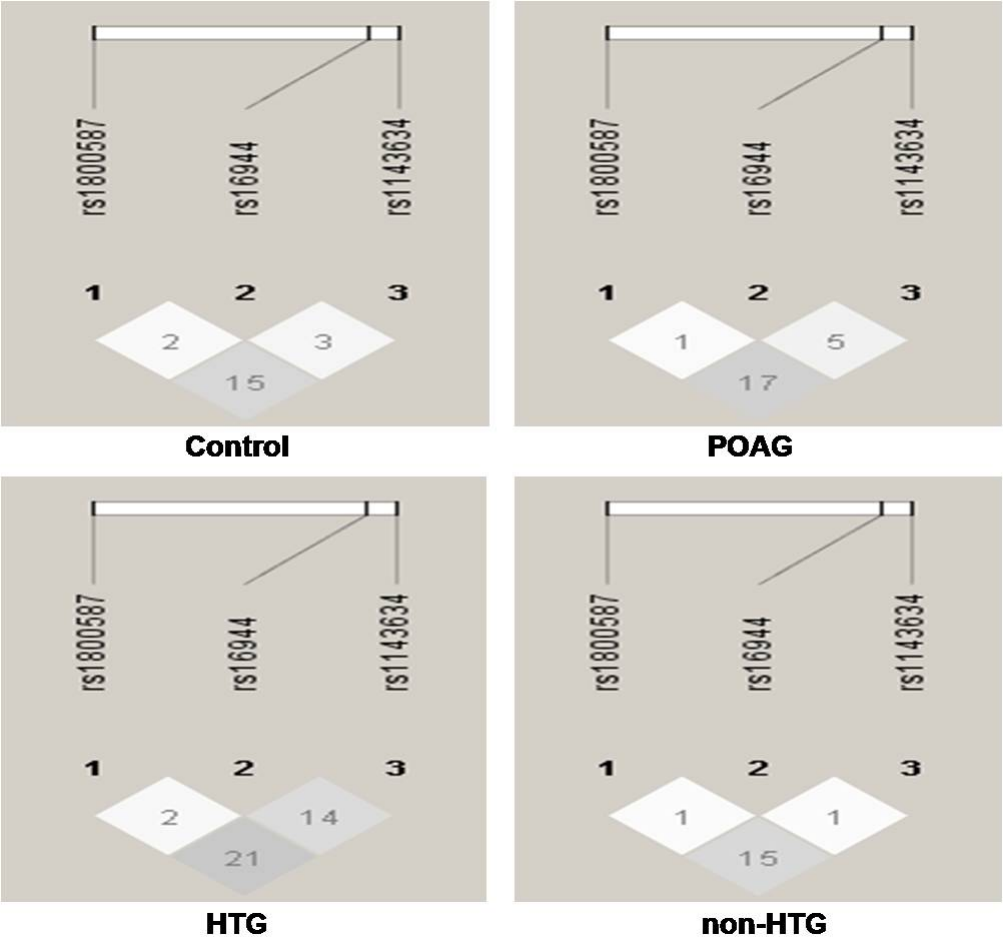
**LD structure between the three SNPs in POAG patients and controls**. No significant LD was observed between the three SNPs. The LD value was calculated using Haploview 4.1 software. The values in the boxes are the r-squared pairwise LD values given by the software. The standard r-squared color scheme is followed here i.e the extent of LD increases with darker shades.

Haplotype analysis revealed presence of total eight haplotypes in both patients and controls. No significant LD was observed between the three SNPs genotyped (Figure 2). Among the eight haplotypes, five (CTC, CCC, TTC, TCT, TCC) represented the majority of chromosomes (~95%) in both POAG patients and controls (Table 2). The remaining three haplotypes (TTT, CTT, CCT) were in much lower frequency (i.e. up to max. 3.5%) and not considered for further analysis as predicted number of patients and controls carrying these haplotypes were too small for carrying out any association study. Thus for haplotypes five tests were considered for Bonferroni correction. Among the five major haplotypes TTC haplotype was significantly under-represented in non-HTG patient group (OR = 0.538.; 95% CI = 0.356-0.815; p = 0.003) as well as the entire POAG (OR = 0.697; 95% CI = 0.498-0.976; p = 0.036) group compared to the control group (Table 2). However after correction for multiple tests by Bonferroni method, the association for POAG group did not sustain. Thus, it appears that the lower p-value for POAG is contributed entirely due to the association with the non-HTG group since no significant association of TTC haplotype was observed with HTG patient group. On the other hand, TCT haplotype was found to be over-represented in HTG patient group (OR = 1.784; 95% CI = 1.084-2.937; p = 0.022) but the association was nullified after correction for multiple tests (Table 2).

**Table 2:** Haplotype distribution in *IL1 *gene cluster between POAG patients and controls

Haplotypes	Patient Subgroup	Frequency in Patients	Frequency in Controls	p-value	Adjusted p-value*	OR (95% CI)
CTC	POAG	0.411(259)	0.423(255)	0.699		
	Non-HTG	0.423(168)		0.963		
	HTG	0.381(88)		0.244	-	-

CCC	POAG	0.264(166)	0.234(141)	0.235	-	-
	Non-HTG	0.28(111)		0.111	-	-
	HTG	0.245 (57)		0.727	-	-

**TTC**	POAG	0.108(68)	**0.146(89)**	0.036	0.18	0.697(0.498-0.976)
	**Non-HTG**	**0.085(34)**		**0.003**	**0.015**	**0.538(0.356-0.815)**
	HTG	0.15(35)		0.912	-	-

TCT	POAG	0.084(53)	0.072(43)	0.406	-	
	Non-HTG	0.059(23)		0.395	-	-
	HTG	0.121(28)		0.022	0.11	1.784(1.084-2.937)

TCC	POAG	0.071(45)	0.081(49)	0.510	-	
	Non-HTG	0.078(31)		0.841	-	
	HTG	0.055(13)		0.211	-	

Haplotype was constructed using SNPs of IL1 gene cluster in the following order:-889 C/T (rs1800587), -511C/T (rs16944) and +3953C/T (rs1143634). TTC haplotype have the potential to act as a protective haplotype in non-HTG patient group. The numbers in parenthesis in 3^rd ^and 4^th ^columns represent numbers of chromosomes in the group. * p-values were adjusted by Bonferroni method.

In an attempt to examine whether the effect of the TTC haplotypes in non-HTG patients is related to age, we divided these patients into two groups: (i) up to 50 years, and (ii) above 50 years. We observed that only the patients above 50 years TTC haplotype appeared to have a protective effect (OR = 0.477; 95% CI = 0.287- 0.792; p = 0.004) (Table 3) even after Bonferroni correction.

**Table 3:** Age based distribution of haplotypes in non-HTG patients and controls

Haplotype	Frequency in Patients	Frequency in Controls	p-value	Adjusted p-value*	OR(95%CI)
**AGE GROUP ≤ 50**					

CTC	0.294 (13)	0.420 (131)	0.115	-	-
CCC	0.342(15)	0.227 (71)	0.100	-	-
TTC	0.160 (7)	0.144 (45)	0.794	-	-

**AGE GROUP > 50**					

CCC	0.277 (98)	0.253 (66)	0.489	-	-
CTC	0.435 (154)	0.417 (109)	0.637		
**TTC**	**0.080 (28)**	**0.152 (40)**	**0.004**	**0.02**	**0.477 (0.287-0.792)**

*P-values were adjusted by Bonferroni method

## Discussion

Glaucoma being a complex disease, understanding of the molecular mechanism underlying its pathogenesis is limited. In addition to the identified genetic loci linked to POAG, other genetic factors e.g. *OPA1*, *Apolipoprotein E*, *CYP1B1*, *E-cahderin*, *OPTC *have been reported to elevate the risk of retinal degeneration characteristic of glaucoma [20,29-32]. However, the interplay of genetic factors and environmental cues causing POAG pathogenesis remains almost unexplored.

The association of the immune system with glaucoma has seemingly conflicting aspects as neuroprotective or neurodestructive. T-cell-mediated immune response may initially be beneficial to limit neurodegeneration. However, a failure to properly control aberrant, stress-induced immune response likely converts the protective immunity to an autoimmune neurodegenerative process that can facilitate the progression of neurodegeneration in some glaucoma patients specially in Normal Tension Glaucoma (NTG) cases [8]. Proliferating cytokines, such as IL1 has been reported [6] to have a role in the immune response in glaucoma patients. The IL1 induces the expression and processing of β-amyloid expression protein (APP), resulting in the increased production of secreted APP and further activation of microglia and overexpression of IL1 [33]. Studies show a higher expression of such microglial cells in glaucomatous optic nerve heads, which may play a role in neurodegeneration [8]. IL1 has been shown to promote optic nerve damage by increasing the synthesis of matrix mellanoproteinase-9 (MMP) in glaucoma mouse model, which mimics some aspects of glaucoma [34,35]. IL1 has also been reported to be involved in increased generation of reactive oxygen species (ROS) [36] and nitric oxide synthesis [37], implicated in retinal ganglion cell damage leading to neurodegeneration.

Previous studies [6,24,25] suggest that single nucleotide polymorphisms (SNPs) in *IL1 *gene cluster could influence glaucoma pathogenesis. Such studies have been carried out so far exclusively in Chinese population groups. For example, Wang *et al *[25] reported that the *IL1A *(-889T) allele is significantly over-represented in HTG patients with an IOP > 21 mmHg but not with NTG cases. Lin *et al *[24] reported a possible association of the *IL1B *(+3953T) polymorphism with POAG in a study carried out in a smaller number of patients which could not be further replicated in recent studies by Wang *et al *[27] in NTG patients and by How *et al *in POAG [23] patients (Table 4). Such variation might be due to overall genome of the population groups under study. However, lack of information about the ethnicity and interrelationship between these populations [23-27] limits further analysis of the data.

**Table 4:** Published reports of *IL1 *gene cluster polymorphisms in Chinese population group

SNPs	Patient (n)	Control (n)	Results	Reference
-889C/T (*IL1A*)				
	156 POAG	167	-889T allele is associated with POAG	Wang *et al*, Mol Vis, 2006
	162 NTG	167	No association	Wang *et al*, J of Glaucoma, 2007
	194 POAG (100 HTG, 94 NTG) and 125 PACG	79	No association	How *et al*, IOVS, 2007

-511C/T (*IL1B*)				
	58 POAG	105	No association	Lin *et al*, Ophthalmologica, 2003
	231 NTG	245	No association	Wang *et al*, Mol Vis, 2007
	194 POAG (100 HTG, 94 NTG) and 125 PACG	79	No association	How *et al*, IOVS, 2007

+3953C/T (*IL1B*)				
	58 POAG	105	+3953T allele is associated with POAG	Lin *et al*, Ophthalmologica, 2003
	231 NTG	245	No association	Wang *et al*, Mol Vis, 2007
	194 POAG (100 HTG, 94 NTG) and 125 PACG	79	No association	How *et al*, IOVS, 2007

We observed weak association of *IL1A *(-889C) allele and *IL1B *(+3953T) allele with non-HTG and HTG patient groups respectively but these associations, if any, did not sustain after Bonferroni correction for multiple tests. However, haplotype analysis based on these 3 SNPs examined, suggests one of the haplotype (TTC) provides protection for non-HTG patients above 50 years of age. This indicates that age also has an effect on this association. It is possible that many patients in our cohort with IOP < 20 mmHg represent NTG but we prefer to describe those as non-HTG cases in the absence of rigorous monitoring of diurnal variations of IOP in all the patients. It appears that apparent association of the *IL1 *haplotype with entire POAG patient group is due to high attributable protection of TTC haplotype only for non-HTG cases.

It would be a worthwhile effort to examine association of these haplotypes in other population groups of India. A recent study carried out by the Indian Genome Variation Consortium shows that the Indian population is divided into 4 distinct linguistic groups - Indo-European, Austro Asiatic, Tibeto-Burman and Dravidian, and there exists high degree inter-population variance in allele frequencies due to their genetic diversity [38]. Among the previous studies in Chinese population, How *et al *reported lack of association of the haplotypes with POAG patients [23]. Lack of required information in other pervious publications [23-27] prevented us from attempting a meta-analysis of the data to examine the effect of these haplotypes on glaucoma pathogenesis.

It has been reported that *IL1A *(-889T) allele upregulates transcription of the gene and thus increases the level of the gene product in Alzheimer's disease [39] as well as the plasma level of IL1B [40]. *IL1B *(+3953T) allele is known to increase the level of IL1B production at least by four fold compared to *IL1B *(+3953C) allele. It is possible that *IL1A *(-889C) allele and *IL1B *(+3953T) allele influence the risk of non-HTG and HTG respectively by altering the expression of the respective proteins [41]. However, since the genomic region represented by specific haplotype (and not just this SNP) is associated with non-HTG cases, it appears likely that other yet unidentified variant might be influencing the course of pathogenesis. We plan to explore the question further by fine mapping the genomic region with additional SNPs and then finding the suspect variant (if any) by deep sequencing the minimum critical region. It is also certainly very important that such studies are replicated in larger and more importantly in additional glaucoma patient cohorts to examine whether the *IL1 *locus has a major effect on glaucoma pathogenesis. Interestingly, the *IL1 *gene cluster (2q13) resides in close proximity to one of the POAG loci (GLC1B at 2cen-2q13), also linked to NTG cases [42]. It would be interesting to explore whether there is more than one genomic region associated with POAG in this segment of chromosome 2. Finally, understanding the role of *IL 1 *in glaucoma pathogenesis and identification of predisposing variants might pave the way for better management of the disease through prevention and treatment as appropriate.

## Conclusion

Our data suggest that *IL1A *and *IL1B *SNPs studied are not associated with POAG. However, haplotype constructed with these SNPs are associated with POAG, specifically in non-HTG patients above 50 years of age, which might be due to additional variants located in the genomic region examined. The observation needs to be further vindicated by similar studies in larger and additional cohort of POAG patients.

## Competing interests

The authors declare that they have no competing interests.

## Authors' contributions

SM, DB, SC and AB carried out the wet lab experiments and also involved in data analysis. SM, DB has prepared the manuscript. IM gave intellectual inputs in statistical analysis of the data. AS led the group of ophthalmologist in Dristi Pradip Eye clinic and selected the patient and control samples used in the study. KR conceived the study, led the group in designing experimental strategies and provided intellectual input for giving final shape of the manuscript. All authors read and approved the final manuscript.

## Pre-publication history

The pre-publication history for this paper can be accessed here:

http://www.biomedcentral.com/1471-2350/11/99/prepub
